# The aquaculture microbiome at the centre of business creation

**DOI:** 10.1111/1751-7915.12877

**Published:** 2017-10-24

**Authors:** Karen K. Dittmann, Bastian B. Rasmussen, Mathieu Castex, Lone Gram, Mikkel Bentzon‐Tilia

**Affiliations:** ^1^ Department of Biotechnology and Biomedicine Technical University of Denmark Kgs. Lyngby Denmark; ^2^ Lallemand SAS Blagnac Cedex France

Twelve per cent of the world's population is currently securing their livelihood partly, or fully, through the fisheries and aquaculture sector (FAO Fisheries and Aquaculture Department, 2016). Most people occupied in this sector rely on wild catches; however, fish stocks are becoming depleted with 90% of stocks being fully or overexploited (FAO Fisheries and Aquaculture Department, 2016). A more productive and sustainable aquaculture sector is needed to meet the sustainable development goals (SDGs) of the UN number 2, 12 and 14 and supply a growing world population, which is expected to reach 10^10^ individuals in approximately 30 years (United Nations, Department of Economic and Social Affairs, Population Division, 2015), with high‐quality protein. The aquaculture sector has, within the past few years, surpassed wild catches in the production of seafood (fish and plants combined; Bentzon‐Tilia *et al*., 2016), and overall employment in the fisheries sector has decreased by approximately one million individuals from 2010 to 2014, while the aquaculture sector saw an increase of 0.1 million individuals. In general, a shift has been seen from 1990, where 83% were employed in fisheries and 17% in aquaculture, to 2014 where 67% were employed in fisheries and 33% in aquaculture (FAO Fisheries and Aquaculture Department, 2016). The sector is projected to increase its output from 74 million tons in 2014 to 102 million tons by 2025, and up to 121 million tons by 2030 (FAO Fisheries and Aquaculture Department, 2016). Furthermore, it was recently suggested that the global biological production potential for marine aquaculture is more than 100 times the current global seafood consumption, thus suitable habitats do not seem to be a limiting factor in the growth of the sector (Gentry *et al*., 2017). Consequently, the industry is faced with a need to significantly increase productivity while at the same time securing both livelihoods and sustainability.

Controlling the microorganisms that are associated with aquaculture systems (i.e. the aquaculture microbiome) has always been essential in high‐intensity rearing of fish. Disease outbreaks caused by pathogenic bacteria are believed to be one of the most serious challenges faced by the aquaculture industry (Meyer, 1991), and consequently, extensive measures are taken to limit the introduction and proliferation of such bacteria in the aquaculture systems. Furthermore, microbial activity in these naturally eutrophied systems may produce unwanted toxic metabolites such as hydrogen sulphide (H_2_S), which is formed when microorganisms reduce sulphate (SO_4_^−^) in anaerobic respiration and which interferes with mammalian respiration. However, microbes may also serve as a solution to an array of these very challenges. In the agriculture industry, microbiome‐based products such as seed coatings that increase nutrient uptake in crops, and which antagonize plant pathogenic soil organisms, are becoming increasingly popular tools to improve productivity in a sustainable manner, and microbiome‐based products may reach a market size comparable to that of chemical agro‐chemicals within a few years (Singh, 2017). The very same technologies that have facilitated this development, for example advances in high‐throughput sequencing and synthetic biology, have been proposed to be key in the sustainable development of the aquaculture industry in the coming years as well (Bentzon‐Tilia *et al*., 2016). However, with a few exceptions, such as studies on recirculating aquaculture systems and fish‐associated microbial communities (van Kessel *et al*., 2011; Llewellyn *et al*., 2014), the aquaculture microbiome has not been characterized to the same degree as its terrestrial counterpart. In contrast, most studies concerning the aquaculture microbiome relies on bacterial isolation and PCR‐based approaches. Hence, the implementation of microbiome‐based products is in its infancy and many practices are still of a ‘hope for the best’ fertilization‐based nature (Moriarty, 1997), where specific functional groups of the aquaculture microbiome are enriched for by adding, for example carbon‐rich substrates. This is the case for most ‘biofloc’ approaches where molasses or an equivalent C‐rich fertilizer is added as a means to increase the C:N ratio and induce the growth of the C‐limited heterotrophic fraction of the aquaculture microbiome, which in turn will remove toxic ammonia (NH_3_) from the rearing water and form bioflocs (Bossier and Ekasari, 2017). Recirculated aquaculture systems (RAS) and biofilters have facilitated the rearing of fish in closed systems with a minimum of water being exchanged with the surrounding environment. This relies on the successful colonization of large‐surface area structures by bacteria such as *Nitrosomonas* spp. and *Nitrospira* spp. that in combination convert NH_3_ to nitrate (NO_3_^−^). Common for these approaches is that they in most cases have relied on modulation of the existing microbiome in the system. However, applications of targeted microbiome‐based products containing a seeding microbial assemblage to aid the heterotrophic assimilation of inorganic nitrogen and/or the nitrification process are now a common practice in intensive tropical pond‐based aquaculture systems (Castex *et al*., 2014). In the case of RAS technology, a similar approach to aid in the colonization of biofilters is highly desirable as it may take up to several months to obtain an efficient microbiome, specifically in marine biofilters (Manthe and Malone, 1987; Gutierrez‐Wing and Malone, 2006). Seeding communities of nitrifiers for pond systems are already available, for example Pond Protect by Novozymes (Table 1), and these have been shown to mitigate increased NH_3_ and nitrite (NO_2_^−^) levels in RAS systems as well (Kuhn *et al*., 2010). Furthermore, nitrification can be coupled with an efficient microbial denitrification process as a powerful tool in the complete removal of nitrogenous compounds from the system, and the development and application of a joined nitrification and denitrification approach for recirculated aquaculture systems, similar to the Aqua Science^®^ concept from Camanor, likely represents an area of potential business development. The commercialization of targeted microbiome‐based products containing living microorganisms, such as seeding microbial assemblages that improve water quality, has been seen for use in aquaria for decades, for example the *BIO‐Spira* product from MarineLand Labs and its predecessors, which like Pond Protect and similar microbiome‐based products for aquaculture systems contain bacterial assemblages that remove ammonia and nitrite. Similar microbiome‐based products for use in conjunction with biofloc technology are also available now. One such product is ShrimpShield by Keeton Probiotics, which facilitates biofloc formation, degradation of sludge as well as microbial removal of NH_3_ and NO_2_^−^ (Table 1). Hence, such microbiome‐based products aim to improve water quality and in some cases remove potential pathogens through, for example, competitive exclusion (Table 1).

**Table 1:** Microbiome‐based products for conditioning of water and pond as well as promotion of a healthy production animal microbiome (feed and feed additives)

Target environment	Company	Product	Purpose	Composition	Reference
Water and pond	AquaInTech	PRO4000X, AquaPro B, AquaPro EZ	Degrade organic matter, reduce ammonia, *Vibrio* reduction	2 Strains of Bacillus – *Bacillus subtilis, Bacillus licheniformis*	1, 2, 3
	Biomin	Aquastar	Stabilize water quality, improve pond bottom quality and support the gut health of fish and shrimp	Formula not publicly available	4
	Keeton Industries	Waste & Sludge Reducer	Improve water and bottom quality, pathogen control	*Bacillus cereus* RRRL B‐30535	5, 6
	Keeton Probiotics	ShrimpShield, PondToss	Degrade organic sludge, improve feed efficiency	Formula not publicly available	7, 8
	Lallemand	Lalsea Biorem	Degrade organic matter, reduce ammonia, pathogen control, stabilize pH	7 specific bacterial strains	9
	Novozymes	Pond Plus	Pathogen control, decomposition of organic substances	Spore forming bacteria	10
	Novozymes	Pond Dtox	Hydrogen sulphide control	*Paracoccus pantotrophus*	11
	Novozymes	Pond Protect	Ammonia and nitrite reduction	*Nitrosomonas eutropha*,* Nitrobacter winogradskyi*	12
Gut microbiome (feed, feed additive)	AquaInTech	AquaPro F	Organic matter degradation, improved digestion of feed	Five strains of bacillus combined	13
	Evonik	EcoBiol	Improve gut health	*Bacillus amyloliquefaciens* CECT 5940	14
	Keeton Probiotics	FeedTreat	Degrade organic sludge and improve feed efficiency	Formula not publicly available	15
	Lallemand	Bactocell^®^	Reduce deformities across fish species, improve gut health across a range of fish and shrimp species	*Pediococcus acidilactici* (MA18/5M)	16, 17
	Rubinum	TOYOCERIN^®^	Promote growth, increase specimen homogeneity, improve intestinal mucosa	*Bacillus cereus *var.* toyoi*	18, 19

References: (1) http://www.aqua-in-tech.com/pro4000x.html; (2) http://www.aqua-in-tech.com/aquapro-b.html; (3) http://www.aqua-in-tech.com/aquapro-ez.html; (4) http://www.biomin.net/en/products/aquastar/; (5) http://keetonaquatics.com/beneficial-microbes/waste-and-sludge-reducer/; (6) Patent ‘US 6878373 B2’; (7) http://keetonaqua.com/products/beneficial-microbes/shrimpshield/; (8) http://keetonaqua.com/products/beneficial-microbes/pondtoss/; (9) http://lallemandanimalnutrition.com/en/asia/products/lalsea-biorem-aquaculture/; (10) http://www.syndelasia.com/aquaculture-probiotics/pond-aquaculture-probiotics-amp-water-manage-26/pond-plus_38; (11) http://ponddtox.com/; (12) http://www.syndelasia.com/aquaculture-probiotics/pond-aquaculture-probiotics-amp-water-manage-26/pond-protect_40; (13) http://www.aqua-in-tech.com/aquapro-f.html; (14) http://animal-nutrition.evonik.com/product/feed-additives/en/products/probiotics/ecobiol/pages/default.aspx; (15) http://keetonaqua.com/products/beneficial-microbes/feedtreat/; (16) http://lallemandanimalnutrition.com/en/asia/products/bactocell-2/; (17) http://www.biomar.com/en/denmark/product-and-species/pike-perch/fry_feeds/; (18) http://www.rubinum.es/en/especies/#acuicultura; (19) http://www.rubinum.es/en/productos/.

Another category of microbiome‐based products that is being developed for the aquaculture industry targets the gut of the animal directly (Table 1), equivalent to the more conventional probiotics for livestock and human consumption. Microbial strains evaluated as probiotics for aquaculture are from many phylogenetic lineages; however, most of them belong to two bacterial phyla, the Firmicutes (e.g. *Bacillus* spp., *Lactobacillus* spp., *Lactococcus* spp. and *Carnobacterium* spp.) and the Proteobacteria (e.g. *Vibrio* spp., *Pseudomonas* spp. and *Shewanella* spp.), while yeasts are rarely studied (Gatesoupe, 2007). The majority of the commercially available probiotic feed and feed additives for aquaculture are based on pure or mixed cultures of lactic acid bacteria and Bacilli (Merrifield *et al*., 2010; Castex *et al*., 2014). This includes Bactocell^®^ (Lallemand; Table 1), which is based on a *Pediococcus acidilactici* strain and is, to the best of our knowledge, the only probiotic registered in Europe for use in aquaculture feed. These bacteria are usually well studied and well known for their positive effect on the human and animal gut microbiome (Cutting, 2011). Furthermore, they are Generally Regarded As Safe (GRAS) or Qualified Presumption of Safety (QPS), which makes it easier to obtain authorization for their use in food and feed products. A natural extension of this type of microbiome‐based products, and a potential new avenue to be explored in aquaculture microbiome business creation, is the controlled colonization of the reared fish from larvae to adult by a microbiome that has the desired functional traits and can act as an infection barrier against pathogens and prevent major economic losses by crashes in the population (De Schryver and Vadstein, 2014).

The successful application of probiotic Firmicutes, originally applied as probiotics for humans or livestock, in aquaculture is fortunate considering the divergent niches in which these probiotics need to establish themselves and function. An avenue of potential new enterprises is to develop similar products based on bacteria of marine origin instead. Marine bacteria including members of the *Roseobacter* group and the *Vibrio* and *Shewanella* genera have been studied extensively for their probiotic potential (Austin *et al*., 1995; Ringø and Vadstein, 1998; Díaz‐Rosales *et al*., 2009; D'Alvise *et al*., 2012; Lobo *et al*., 2014; Grotkjær *et al*., 2016; Bentzon‐Tilia and Gram, 2017). Furthermore, these are often found as part of the indigenous microbiome of marine eukaryotes, and although their application as probiotics has been proposed, they have not yet reached a commercialization stage. To succeed with this approach, much more thorough characterizations of aquaculture and marine host microbiomes are needed. Furthermore, in most cases, the putative probiotic candidates reported in scientific publications do not go on to commercialization and industrial application. Getting a probiont to the commercial market requires many additional steps including assessments of safety, scale‐up efficacy, production scale‐up and pre‐market registration. Consistency, efficiency and most importantly safety are key points in all large‐scale productions, and they should be considered from the early stages of the discovery phase to the final application in feed products. Thus, not only does the development of a commercial product rely on substantial financial investments, but also on the contribution from a multidisciplinary team encompassing close collaborations between scientists, aquaculture experts, fermentation engineers and regulatory personnel. The latter part of the team is important for success in a regulatory landscape which varies from an absence of regulation in certain countries to a rigid regulatory framework not always adapted to the effect a probiotic can display. Despite these challenges, the aquaculture industry has already embraced the industrial application of microbiome‐based products for the last two decades, and this has truly created a vast range of new enterprises especially in South East Asia, Central and South America and more recently in Europe.

Using microbiome‐based products also requires developments of production, packaging and distribution technology. One must consider that the efficiency of such products only in part depends on the choice of the microbial strains that compose it (selection), but also on the way the product is produced, conditioned and finally packaged to withstand a variety of storage conditions.

In conclusion, the aquaculture industry is one of the fastest growing food producing sectors in the world and the increased productivity of this sector is essential for the fulfilment of the sustainable development goals of the UN. Microbiome‐based products for application in industrial aquaculture are today a reality, but the full potential is far from exploited. Despite decades of experience and an increasing number of microbial biotechnological products, there is a large innovation potential; from the discovery of new probionts of marine origin and large‐scale cultivation strategies to manoeuvering the political, regulatory landscape and disseminating the use of probiotics to ensure future, sustainable technologies for high‐quality protein production.
